# Ethnic differences in COVID-19 mortality in the second and third waves of the pandemic in England during the vaccine roll-out.

**DOI:** 10.23889/ijpds.v7i3.1929

**Published:** 2022-08-25

**Authors:** Matt Bosworth, Vahé Nafilyan

**Affiliations:** 1 Office for National Statistics

## Objectives

This study aims to assess whether ethnic differences in COVID-19 mortality in England have continued into the third wave and to what extent differences in vaccination rates contributed to excess COVID-19 mortality after accounting for other risk factors.

## Approach

This Cohort study of 28.8 million adults living in private households or communal establishments in England is based on data from the Office for National Statistics (ONS) Public Health Data Asset (PHDA). The ONS PHDA is a linked dataset combining the 2011 Census, mortality records, the General Practice Extraction Service (GPES) Data for Pandemic Planning and Research (GDPPR), Hospital Episode Statistics (HES) and vaccination data from the National Immunisation Management System (NIMS). We calculated hazard ratios (HRs) for death involving COVID-19 during the second (8 December 2020 to 12 June 2021) and third wave (13 June 2021 to 1 December 2021) of the pandemic separately for males to females to assess the association between ethnic group and death involving COVID-19 in each wave, sequentially adjusting for age, residence type, geographical factors, sociodemographic characteristics, pre-pandemic health, and vaccination status.

## Results

Age-adjusted HRs of death involving COVID-19 were higher for most ethnic minority groups than the White British group during both waves, particularly for groups with lowest vaccination rates (Bangladeshi, Pakistani, Black African and Black Caribbean). In both waves, HRs were attenuated after adjusting for geographical factors, sociodemographic characteristics, and pre-pandemic health. Further adjusting for vaccination status substantially reduced residual HRs for Black African, Black Caribbean, and Pakistani groups in the third wave. The only groups where fully-adjusted HRs remained elevated were the Bangladeshi group (men: 2.19, 95% CI 1.72 to 2.78; women: 2.12, 95% CI 1.58 to 2.86) and men from the Pakistani group (1.24, 95% CI 1.06 to 1.46).

## Conclusion

Public health strategies to increase vaccination uptake in ethnic minority groups could reduce disparities in COVID-19 mortality that cannot be accounted for by pre-existing risk factors.

